# Fear of Cancer Recurrence and Associated Factors Among Women with Female Cancer: A Cross-Sectional Study

**DOI:** 10.3390/healthcare14172722

**Published:** 2026-08-26

**Authors:** Lovorka Brajković, Ivana Lozić, Dora Korać, Vanja Kopilaš

**Affiliations:** Department of Psychology, Faculty of Croatian Studies, University of Zagreb, Borongajska cesta 83d, 10000 Zagreb, Croatia; lbrajkov1@fhs.unizg.hr (L.B.); ivana.lozic4@gmail.com (I.L.); vkopilas@fhs.unizg.hr (V.K.)

**Keywords:** fear, cancer survivor, breast cancer, gynecological cancers, self-efficacy

## Abstract

**Background/Objectives**: The emotional impact of a cancer diagnosis may become particularly salient during survivorship, as survivors continue to face fears and concerns related to recurrence. Fear of cancer recurrence (FCR) is one of the most prevalent psychological challenges and significant unmet need among women with female cancer. The aim of this study was to assess the presence of FCR in predominantly breast cancer survivors, and to examine its relationship with certain demographic, clinical, and psychosocial factors. **Methods**: This cross-sectional study was conducted with a convenience sample of 120 women with a history of breast or gynecological cancer who were recruited over a two-month period through several cancer support associations across Croatia. Data were collected in person using a questionnaire comprising sociodemographic and clinical variables, as well as four self-reported instruments: the Fear of Cancer Recurrence Inventory–Short Form, Impact of Event Scale, Cancer Behavior Inventory–Brief Version, and Multidimensional Scale of Perceived Social Support. Data were analyzed using descriptive statistics, Pearson’s correlation coefficient and hierarchical regression analysis. **Results**: In the final sample of 110 women, FCR levels were moderate, with 66.4% of participants reporting clinically significant levels. FCR was positively associated with chemotherapy (*r* = 0.22, *p* < 0.05), intrusive thoughts (*r* = 0.56, *p* < 0.01) and avoidance tendencies (*r* = 0.39, *p* < 0.01), and negatively associated with age (*r* = −0.26, *p* < 0.01) and self-efficacy for coping (*r* = −0.41, *p* < 0.01). In the final regression model, intrusive thoughts, coping self-efficacy, and chemotherapy were the only variables independently associated with fear of cancer recurrence. **Conclusions**: These findings indicate the presence of FCR in the period after primary treatment, particularly among breast cancer survivors, and highlight cognitive processing of cancer-related experiences and self-efficacy as potentially relevant areas for further research.

## 1. Introduction

With an estimated 2.3 million newly diagnosed cases reported in 2022 [1], and 3.2 million cases projected by 2050, breast cancer remains a major public health concern [2]. According to recent global statistics, breast cancer is the most frequently diagnosed cancer among women and the second most commonly diagnosed cancer worldwide, closely following lung cancer [1,3]. It accounts for nearly one third of all cancer cases and remains the leading cause of cancer-related death among women. Alongside breast cancer, gynecological malignancies, such as cervical and endometrial cancers, also represent a substantial health burden among women worldwide. In 2022, cervical cancer was the fourth most commonly diagnosed cancer and the fourth leading cause of cancer-related mortality among women globally [4]. At the European Union level, breast and gynecological cancers are among the four most common cancer sites in women. This pattern is consistent with trends observed in Croatia, where these malignancies are also among the five leading causes of cancer-related mortality [5]. Exploring breast and gynecological cancers together may hold promise, as they often share biological drivers, genetic predispositions like BRCA mutations, and hormonal pathways that could facilitate new medical breakthroughs [6,7,8].

Although advances in early detection and treatment have substantially improved survival rates among women diagnosed with breast and gynecological cancers [9], the completion of primary treatment does not necessarily signify the end of the cancer experience. Rather, the period following the end of primary treatment represents a complex phase characterized by the need for psychological, physical, and social adjustment [10]. Alongside continuous monitoring of their physical health, women are often expected to gradually readjust to their “pre-cancer” lives and resume family, social, and occupational roles, while simultaneously coping with the potential long-term consequences of cancer treatment. Although no longer undergoing active treatment, many women remain in ongoing oncological follow-up, which commonly includes regular hormonal therapy use aimed at recurrence prevention and routine medical examinations [11]. Cancer survivorship begins at the time of diagnosis and continues throughout the entire disease continuum, encompassing active treatment, advanced disease, remission, and long-term follow-up [12]. However, the psychosocial challenges that arise following treatment often receive insufficient recognition and attention, as survival outcomes tend to remain the primary focus of care [13]. Consequently, many women continue to experience significant psychological distress, including depression, anxiety, stress, and fear of cancer recurrence [14,15,16]. Fear of cancer recurrence (FCR), often described as the fear or concern that cancer may return or spread to the same or another part of the body, is considered one of the most prevalent psychological challenge among oncology patients and survivors [16,17]. Previous studies have reported that the prevalence of FCR ranges between approximately 40% and 75%, with a substantial proportion of women experiencing clinically significant levels of fear that negatively affect daily functioning [18,19,20]. Longitudinal findings further suggest that moderate levels of FCR remain relatively stable for up to 5 years following the completion of active treatment [21,22,23]. Additionally, some studies have documented its persistence even ten years after diagnosis [24].

**Figure 1:**
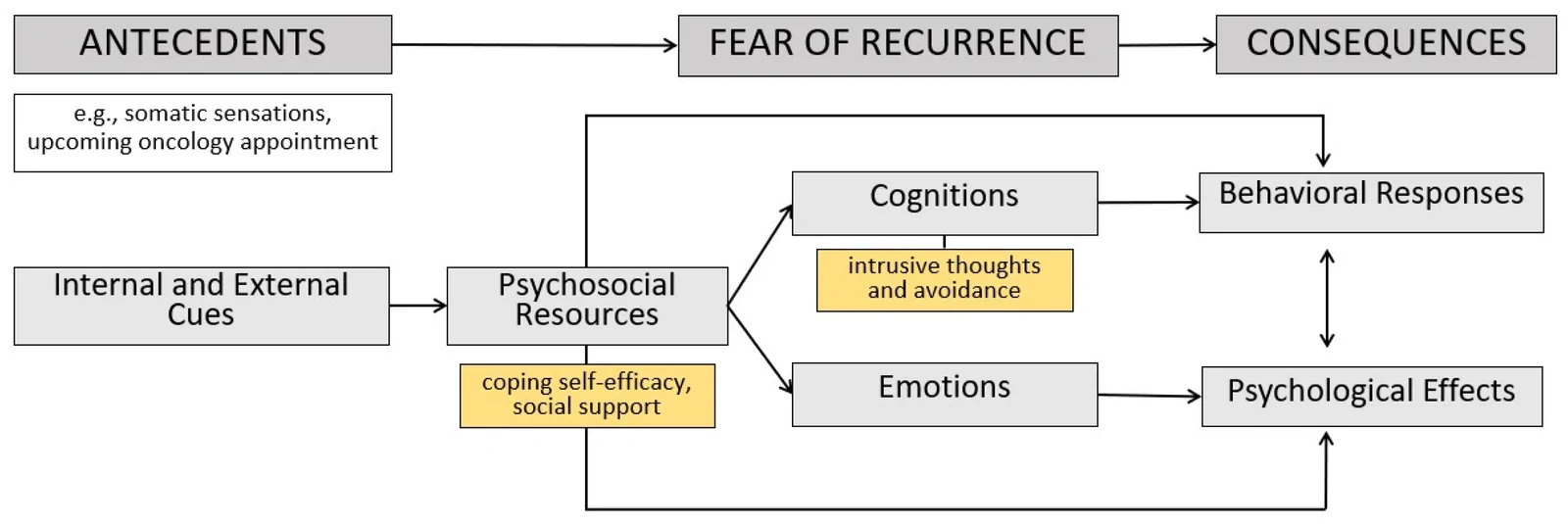
Lee-Jones model of fear of cancer recurrence.

In order to better understand the mechanisms underlying the development of FCR, Lee et al. [25] proposed the Lee-Jones model of fear of cancer recurrence (Figure 1). According to this model, FCR is conceptualized as a dynamic process in which antecedent individual and contextual factors, including age, treatment type, severity of somatic symptoms (e.g., fatigue and nausea), coping strategies, contribute to its development and maintenance through cognitive and emotional processing mechanisms. Younger women have consistently been found to report higher levels of FCR, with younger age emerging as a significant predictor of its severity [16,18,26]. Shorter time since diagnosis, chemotherapy, and upcoming oncological examinations could also contribute to elevated FCR by increasing vulnerability to maladaptive cognitive–emotional processing characterized by intrusive thoughts, worry, rumination, and avoidant behaviors [21,27]. Cancer survivors who exhibited lower tolerance for uncertainty and reported higher levels of rumination experienced greater FCR [28]. Additionally, the presence of more frequent intrusive thoughts and elevated pathological worry was associated with increased fear of cancer recurrence [29,30]. Women with moderate to high FCR reported greater use of distraction and cognitive avoidance compared with those with lower FCR [31,32]. Within the Lee-Jones model [25], these cognitive processes are considered central mechanisms underlying the development and maintenance of FCR. In particular, intrusive thoughts have been identified as a strong predictor of fear of recurrence among both cancer survivors undergoing active treatment and those in the post-treatment period [33]. In the extended model of FCR, Lucas et al. [34] further introduced self-efficacy for coping as an important protective factor in psychological adaptation to cancer. It reflects individuals’ perceived ability to effectively manage disease-related symptoms, emotional distress, and treatment-related challenges. Consistent with the model, self-efficacy for coping with cancer could facilitate more adaptive interpretations of threatening cancer-related stimuli and promote more effective emotional responses, including fewer intrusive thoughts and lower levels of avoidance [34]. Consequently, higher coping self-efficacy may mitigate FCR and facilitate better functioning among cancer survivors [26,28,35].

Although social support is not explicitly incorporated within the Lee-Jones model, its importance in post-cancer psychological adjustment has been widely recognized [10,36]. Social support from family, friends, and healthcare professionals is among the most commonly reported unmet supportive care needs among breast cancer survivors [15]. Given its important role in reducing FCR, adequate social support represents a key component of survivorship care [37]. Emotional and instrumental support provided by significant others has consistently emerged as a significant predictor of lower levels of FCR among women diagnosed with breast cancer [18,19,38]. Both perceived and objectively received social support independently contribute to lower levels of FCR by facilitating more adaptive cognitive–emotional processing [39]. In this context, social support may reduce illness-related uncertainty and strengthen psychological resilience, which in turn may contribute to lower FCR [36,37].

Given the presence and persistence of fear of recurrence among cancer survivors, as well as its association with depression, anxiety, quality of life and long-term adjustment following treatment [15], it is essential to further investigate the contextual and individual factors contributing to its severity. Therefore, the present study aims to examine the level of fear of cancer recurrence among breast and gynecological cancer survivors in Croatia, where research on this topic remains scarce. Building on previous findings, the study also aims to investigate associations between FCR and certain demographic, clinical, and social factors, including age, chemotherapy, time since diagnosis, time since the most recent follow-up examination, and perceived social support. Additionally, the study observed coping self-efficacy and cognitive processing of cancer-related experiences, as one of the key components of the Lee-Jones model of FCR. It was hypothesized that higher levels of FCR would be associated with younger age, shorter time since diagnosis, greater intrusive thoughts and avoidance tendencies, lower coping self-efficacy, and lower perceived social support, and that these variables would be independently associated with FCR.

## 2. Materials and Methods

### 2.1. Study Design

A cross-sectional study was conducted among members of seven associations for women with a history of breast or gynecological cancer.

### 2.2. Participants

An a priori power analysis was conducted using G*Power version 3.1.9.7. Assuming a medium effect size of 0.15, a statistical power of 0.80, and a significance level of 0.05, the analysis indicated that a minimum of 103 participants was required for a multiple regression analysis with seven independent variables. To account for potential incomplete responses, the sample size was increased by approximately 10%, resulting in a minimum target sample of 113 participants.

The inclusion criteria were as follows: (1) women aged 18 years or older; (2) self-reported previous diagnosis of breast and/or gynecological cancer; (3) and completed primary cancer treatment such as surgery, radiotherapy, chemotherapy (but not hormonal therapy). Participants were excluded if they were currently undergoing treatment for breast or gynecological cancer (surgery, radiotherapy, chemotherapy), reported a psychiatric disorder or had missing data on the observed variables.

### 2.3. Instruments

#### 2.3.1. Sociodemographic Questionnaire

A structured questionnaire was used to gather sociodemographic information including gender, age, children, educational level, marital status, and employment status. These were followed by questions related to the cancer diagnosis (e.g., disease stage and time since diagnosis), treatment history (e.g., surgical procedures and received primary treatment), and current health status (e.g., remission status and time since the most recent medical examination).

#### 2.3.2. Fear of Cancer Recurrence Inventory—Short Form (FCRI-SF)

Fear of Cancer Recurrence Inventory—Short Form (FCRI-SF [40]) is a unidimensional scale that consists of 9 items. Using a 5-point Likert scale ranging from 0 (“Not at all”) to 4 (“A great deal”), participants indicated the extent to which they experienced fear or concern related to the possibility of cancer recurrence during the past month. Total scores on the scale range from 0 to 36, with higher scores indicating greater severity of FCR. The Croatian translation of the FCRI-SF previously used in a sample of breast cancer survivors was administered in the present study [41]. According to Simard and Savard [40], a score of 13 or higher represents the optimal cut-off for identifying clinically significant fear of recurrence, while a score of 16 or higher may be used as a cut-off for identifying potentially pathological levels. FCRI-SF showed good internal consistency with Cronbach’s alpha of 0.89 in the original study [40], and 0.77 in a Croatian sample [41]. The one-factor structure was examined, with most factor loadings ≥0.30 and one item showing a lower loading. The item was retained in the total score, consistent with the original scale structure. The Cronbach’s alpha obtained in the present study (α = 0.85) indicates good internal consistency.

#### 2.3.3. Cancer Behavior Inventory–Brief Version (CBI-B)

Self-efficacy for coping with cancer was assessed using the Cancer Behavior Inventory–Brief Version (CBI-B [42]). It consists of 12 items distributed between four subscales: (1) Maintaining independence and positive attitude; (2) Participating in medical care; (3) Coping and stress management; and (4) Managing affect. The original CBI-B was adapted for use in Croatian using a translation and back-translation procedure conducted by two independent experts. The content appropriateness of the items was evaluated by third expert with extensive experience in working with oncology patients. Exploratory factor analysis of the Croatian version yielded the expected four-factor structure, with factor loadings ≥0.30. Participants rated their confidence in managing various aspects of coping with cancer on a Likert-type scale ranging from 1 (not at all confident) to 9 (totally confident). The total score is calculated by summing up estimates on all items, with higher scores indicating higher self-efficacy for coping with cancer. Previous Cronbach’s alpha values (α from 0.84 to 0.89) indicate good reliability [42,43], which is confirmed in the present study (α = 0.87).

#### 2.3.4. Impact of Event Scale (IES)

Impact of Event Scale (IES [44]) translated and validated in Croatian [45], was used to assess subjective distress associated with a specific life event, in this case, the diagnosis of breast and/or gynecological cancer. It consists of 15 items divided into two subscales, Intrusion (7 items) and Avoidance (8 items), scored on a 5-point Likert scale ranging from 0 (never) to 4 (often). Total scores are calculated by summing item responses, with higher scores indicating greater subjective distress related to the cancer diagnosis, reflected in a higher presence of intrusive thoughts and feelings as well as greater tendencies toward avoidance. Previous studies reported good internal consistency for both the overall scale (Cronbach’s α = 0.86) and its subscales (α = 0.78 for Intrusion and α = 0.82 for Avoidance; [44]). Cronbach’s α coefficients ranging from 0.79 to 0.92 have been previously reported in a Croatian sample [46]. In the present study, Cronbach’s α values were 0.90 for the total scale, 0.88 for the Intrusion subscale, and 0.84 for the Avoidance subscale.

#### 2.3.5. The Multidimensional Scale of Perceived Social Support (MSPSS)

The Croatian translation [47] of Multidimensional Scale of Perceived Social Support (MSPSS [48]) was used to assess perceived social support from family members, friends and significant others. Participants rated each of the 12 statements using a 7-point Likert scale ranging from 1 (strongly disagree) to a 7 (strongly agree). Total score on all three subscales is calculated as an average value of responses, with higher scores indicating greater perceived support. The three-factor structure was examined, with factor loadings ≥0.30 observed across all factors. The subscales show excellent reliability with Cronbach’s alpha above 0.92 [47] and above α = 0.93 in this study.

### 2.4. Ethical Considerations

The study was conducted in accordance with the Declaration of Helsinki and approved by the Ethics Committee of Department of Psychology at the University of Zagreb Faculty of Croatian Studies (protocol code: 053-01/25-2/0001).

Prior to data collection, the aim of the study, research procedures, and participants’ rights were explained both verbally and in written form. All participants provided written informed consent, confirming their understanding of the study purpose and their right to withdraw without consequences. All research procedures conducted within the participating associations were carried out in the presence of the researchers, who are also authors of this study. In addition, participants were informed that confidentiality of the data would be ensured through the use of self-generated unique identification codes. Completed paper questionnaires were stored in a secure locked location, while electronic data stored in a password-protected folder accessible only to the authors of the study. Participation was entirely voluntary, and the participants received no monetary compensation for the participation.

### 2.5. Data Collection

During March and April, 16 associations providing support to women with a history of breast and/or gynecological cancer were identified and contacted regarding their current activity and potential interest in participating in the study. The study included members of seven associations from different cities across Croatia. Following ethical approval, representatives of participating associations distributed the invitation to participate, along with information regarding the time and location of data collection, to their members. The data collection was conducted in person at the conference rooms of the participating associations, over a two-month period, from 12 May to 26 June 2025.

### 2.6. Data Analysis

The dataset was analyzed using the IBM SPSS Statistics program (version 26). Significance was set at *p* < 0.05.

The Shapiro–Wilk normality test identified deviations from the normality of distribution for most of the variables which were somewhat expected considering that data was collected on a small clinical sample. Descriptive statistics were conducted to determine the mean and standard deviations for observed continuous variables. For variables with asymmetric distributions, both the mean and median values are reported. Categorical demographic and clinical variables, such as educational level, stage of cancer and treatment type, are presented as frequencies.

Considering the sample size, and the adequacy of the distributional properties (within skewness ± 3; kurtosis ± 10 [49]), Pearson correlation analysis was conducted to examine the relationships between fear of cancer recurrence and certain demographic, clinical, and psychosocial factors (intrusive thoughts, avoidance, self-efficacy for coping, perceived social support).

To examine whether the selected demographic, clinical, and psychosocial variables were independently associated with FCR among women diagnosed with breast and/or gynecological cancer, a hierarchical multiple regression analysis was conducted. The variables entered in the first step represented demographic and individual clinical characteristics that have been reported as potentially relevant to FCR, including age, time since diagnosis, and chemotherapy. The subsequent blocks included variables reflecting relevant concepts within the selected FCR model. Intrusive thoughts and avoidance were included in the second block, while coping self-efficacy and perceived social support were included in the third block. Variables were entered at each step using the Enter method. Prior to conducting the hierarchical regression analysis, multicollinearity was assessed using the Variance Inflation Factor (VIF < 10) and Tolerance values (>0.20). As all values were within acceptable limits (VIF from 1.04 to 1.86; Tolerance from 0.54 to 0.96), no violations of the multicollinearity assumption were detected. Furthermore, for smaller samples Cook’s distance values below 1 and Mahalanobis distance values below 15 indicate the absence of multivariate outliers [50]. Based on these criteria, participants with values of Mahalanobis distance exceeding 15 and those with missing data for the clinical variable considered in the regression model (time since diagnosis) were not included in the analysis.

This study was reported in accordance with the Strengthening the Reporting of Observational Studies in Epidemiology (STROBE) guidelines for cross-sectional studies (see Supplementary Materials).

## 3. Results

### 3.1. Participant’s Characteristics

Seven cancer support associations from different cities across Croatia responded to the invitation to participate, with an estimated total membership of approximately 900 women with a history of breast or gynecological cancers. As invitations were distributed by association representatives, the exact number of women invited to participate could not be determined. Following the invitation, which included the study eligibility criteria, 120 members participated in the in-person data collection. Ten women were excluded from further analyses due to partially or fully missing data on the psychological measures, resulting in a final sample of 110 participants, which was slightly below the initially targeted sample size. Among them, 96 women had been diagnosed with breast cancer, 13 with gynecological cancers, and one woman had been diagnosed with both breast and gynecological cancer. The age range was between 31 and 87 years, with an average age of 61 years (*M* = 61.3, *SD* = 11.6). The majority of observed cancer survivors were married (57.3%), had children (90.9%), and were retired at the time of participation in the study (57.3%).

With regard to clinical characteristics of the treatment process, the largest proportion of women (48.2%) had been diagnosed with Stage I cancer. In addition, 70.9% had no metastases at the time of diagnosis, and 51.8% reported no family history of breast or gynecological cancers. On average, almost nine years had passed between the time of diagnosis and participation in the study (in months *M* = 104.3, *SD* = 99.7), with the time since diagnosis ranging from 2 months to 41 years. Participants often received a combination of treatment modalities. The three most commonly reported treatment modalities were surgery (86.4%), chemotherapy (60.0%), and radiotherapy (56.4%). Regarding current health status, the majority of women (65.5%) were in remission from breast or gynecological cancers, while 49.1% continued to receive hormonal therapy as part of their treatment at the time of the study. On average, approximately nine months had elapsed since participants’ most recent oncologist visit (*M* = 8.7, *SD* = 16.3). Additional sample characteristics are presented in Table 1.

### 3.2. Descriptive Statistics of Study Variables

Table 2 shows descriptive data for FCR, subjective distress (total score and for subscales of intrusion and avoidance), self-efficacy for coping and perceived social support (total score and for all three subscales). In terms of FCR, participants reported a moderate mean level of fear of cancer recurrence (*M* = 15.3, *SD* = 7.7). Based on established cut-off scores [40], a score of ≥13 indicates clinically significant fear of recurrence, while ≥16 reflects severe or pathological levels. In the present study, 66.4% of women (*n* = 73) screen positive for clinically significant FCR (≥13), and 47.3% (*n* = 52) reached the ≥16 cut-off. On average, participants reported moderate levels of subjective stress (*M* = 27.7, *SD* = 16.8). Avoidance-related responses to the diagnosis (*M* = 16.9, *SD* = 9.95) were somewhat more prevalent than intrusive thoughts (C = 8, range = 0–33). They have also demonstrated a relatively high level of self-efficacy for coping with cancer, as reflected in the mean CBI-B score (*M* = 86, *SD* = 15.5). Additionally, the observed mean scores and score distributions indicate relatively high levels of perceived social support from family, friends, and significant others.

### 3.3. Correlation Among Observed Variables

To determine the association between observed variables, Pearson’s correlation analysis was conducted (Table 3). A significant positive association was established between FCR and cognitive–emotional processing, specifically intrusive thoughts and avoidance-related responses. Cancer survivors who reported greater FCR tend to experience more frequent intrusive thoughts related to their cancer diagnosis and avoidant patterns of thinking and behavior. FCR was negatively associated with age and self-efficacy for coping, indicating that older participants and those reporting higher confidence in their ability to cope with treatment-related challenges, side-effects experienced lower levels of FCR. Regarding clinical treatment characteristics, a significant association was observed only for chemotherapy, with higher levels of FCR observed among cancer survivors who received chemotherapy as part of their treatment. No statistically significant relationship was found between FCR and perceived social support. Furthermore, higher self-efficacy for coping was associated with lower levels of intrusive thoughts and avoidance. Higher levels of coping self-efficacy were associated with greater perceived social support from family and significant others. Additionally, negative correlations were observed between perceived social support and avoidance behaviors.

### 3.4. Predicting Fear of Cancer Recurrence

The hierarchical regression analysis was conducted on 102 women due to missing data for the variable time since diagnosis and Mahalanobis distance values exceeding the specified threshold of 15 [50]. In the first step, age and clinical variables were entered into the model. Intrusive thoughts and avoidance were added in the second step, followed by coping self-efficacy and perceived social support in the final step.

The hierarchical regression analysis (Table 4) indicated that each of the three blocks were significant (*F*_Model1_ = 4.62; *p* < 0.001; *F*_Model2_ = 13.96; *p* < 0.001; *F*_Model3_ = 11.830; *p* < 0.001). The first block explained 12.3% of the variance (*R*^2^ = 0.123; *R*^2^adj = 0.096; *p* < 0.01), while the second block explained 41.8% (*R*^2^ = 0.418; *R*^2^adj = 0.388; *p* < 0.01) of the variance of fear of cancer recurrence. With the inclusion of coping self-efficacy and perceived social support in the third block, the model explained 46.6% (*R*^2^ = 0.466; *R*^2^adj = 0.426; *p* < 0.01) of the variance. In the final model, chemotherapy (β = 0.242, *p* = 0.002), intrusive thoughts (β = 0.498, *p* < 0.001) and self-efficacy for coping (β = −0.203, *p* = 0.03) were independently associated with FCR.

## 4. Discussion

Given the significant implications of FCR for survivors’ quality of life and their physical, psychological, and social adjustment following active cancer treatment, investigating its levels within the sample and identifying associated demographic, clinical and psychosocial factors remain an important area of research. Findings of this study were partially consistent with the Lee-Jones model and previous research on FCR. FCR was significantly associated with younger age, intrusive thoughts, avoidance tendencies, and lower coping self-efficacy, but not with time since diagnosis or perceived social support. Of the variables included in the analysis, only chemotherapy, intrusive thoughts, and coping self-efficacy emerged as independent correlates of FCR.

Although the average level of FCR was within the moderate range, more than half of the included women screen positive for clinically significant FCR (≥13), with around 47% screen positive for severe or pathological levels, according to FCRI-SF screening threshold [40]. The levels of FCR observed in the present study align with those reported in previous studies involving cancer survivors [16,18,51]. Similar findings have recently been reported among breast cancer survivors [52]. Nevertheless, studies focusing on younger women with advanced-stage breast cancer diagnosed within the preceding five years have identified substantially higher levels of FCR [51]. Additionally, somewhat higher levels of FCR were observed among gynecological cancer survivors [53]. Lower levels found in this study could be attributable to differences in sociodemographic and clinical characteristics across study samples, including age, cancer type, disease stage, and time since diagnosis. Specifically, the sample in this study was predominantly composed of women with early-stage disease, whereas women with gynecological cancers constituted a smaller proportion of the participants. Additionally, this study included somewhat older participants, with an average of almost nine years having elapsed since diagnosis. Previous research has suggested that older age may represent a potential protective factor against higher FCR severity, with higher levels of FCR observed among women with a shorter time since diagnosis [24,54]. Consistent with previous research, younger age was associated with higher levels of FCR in the present study [26,55]. However, age was not identified as a significant associated factor of FCR levels in the final model. This finding should be interpreted in light of the clinical characteristics of the present sample, as previous studies have included a greater proportion of women with more advanced disease stages [38]. Women who had undergone chemotherapy reported higher levels of fear of cancer recurrence, which is in line with existing literature [20,39]. However, in this study, neither time since diagnosis nor time since the most recent medical examination was related to FCR. This finding is somewhat consistent with the Lee-Jones model, which conceptualizes FCR as a persistent and ongoing fear that may remain present regardless of the time elapsed since diagnosis [25,56]. Previous correlational and longitudinal studies have reported that FCR remains a salient concern among long-term breast cancer survivors and that fear levels remain relatively stable throughout the first 18 months following diagnosis [16,26]. Additionally, women in this study were, on average, almost nine years post-diagnosis and most of them had been diagnosed with early-stage disease. The theoretical model further proposes that FCR may temporarily increase in anticipation of follow-up examinations, such as mammography [25,34]. As noted above, no association was found between the time elapsed since the most recent follow-up examination and FCR. One possible explanation for this finding is that participants, on average, reported a somewhat longer interval since their last follow-up examination than would be expected to capture short-term fluctuations in FCR.

Furthermore, cancer survivors included in this study, who felt more confident in their ability to manage emotional distress and treatment-related side effects reported lower levels of fear of recurrence. This finding is consistent with previous research indicating that greater coping self-efficacy is associated with lower levels of clinically significant FCR [31]. This negative association has been consistently observed, across different cancer stages and types of malignancy [55]. It is important to consider that the sample in this study predominantly comprised breast cancer survivors. Among breast cancer patients, greater coping self-efficacy has also been associated with greater perceived controllability of the illness, which may be related to lower levels of FCR [26]. Consistent with the revised Fear of Cancer Recurrence model [34], this may partly reflect the role of coping self-efficacy in shaping cognitive–emotional processing. Individuals who perceive themselves as capable of managing treatment-related side effects, regulating stress and negative affect, and actively engaging in treatment decisions could experience fewer intrusive thoughts and less avoidance. In line with previous literature, women in this study with greater confidence in their ability to cope with cancer-related challenges also reported fewer intrusive thoughts, less reliance on avoidance-based coping strategies, and greater perceived social support. The positive association between coping self-efficacy and social support has been confirmed among individuals with various malignancies [57]. Furthermore, social support has been identified as an important resource for promoting physical and psychosocial adjustment following cancer treatment and has consistently been associated with lower levels of FCR [58]. Subjective support in terms of understanding and emotional support from family and friends was associated with reduced FCR among women with different stages of breast cancer [18,37]. However, contrary to expectations, perceived social support was not significantly associated with FCR in the present study. This finding may be partly attributable to the generally high levels of social support reported by participants on the self-report measure, which may have limited variability. Additionally, as this study included only women recruited through cancer support associations, participants could have had greater access to instrumental, emotional, and informational support.

Regarding the cognitive mechanisms underlying fear of cancer recurrence, intrusive thoughts emerged as the strongest independent correlate. The role of cognitive processing in FCR during the post-treatment period is supported by previous research and reflected in the Lee-Jones model [25,59]. Intrusive thoughts were a significant predictor of FCR among individuals in both active treatment and post-treatment survivorship phases [33]. Women with breast cancer who report clinically significant levels of FCR also tend to experience more frequent intrusive thoughts, greater intolerance of uncertainty, and higher levels of worry [30]. The significant contribution of intrusive thoughts to FCR is further supported by recent qualitative findings, in which intrusive thoughts emerged as a major source of psychological distress and an important trigger of recurrence-related fears among individuals with various cancer diagnoses [27]. However, the observed moderate association between intrusive thoughts and FCR should also be considered, as it may reflect a certain degree of overlap between the constructs. This overlap may be partly related to the use of self-report measures, as some items assessing these constructs share similar content.

### Limitations and Future Directions

The present study contributes to the understanding of fear of cancer recurrence among survivors of breast and gynecological cancers, particularly regarding the role of individual psychological resources and cognitive processes associated with FCR, providing further support for some of the assumptions of the Lee-Jones model [25]. Nevertheless, several limitations of the present study should be acknowledged when interpreting the findings. Due to the cross-sectional design, it is not possible to establish temporal or causal relationships between FCR and observed clinical and psychosocial variables. Participants were recruited through cancer support associations, which may limit the generalizability of the findings due to potential selection bias. It should also be noted that participants completed the questionnaires in small groups, which may have increased the potential for socially desirable responding. In addition, participation bias cannot be ruled out, as women engaged with cancer support associations may have been more likely to participate in the study. They may also differ from the overall population of breast and gynecological cancer survivors with respect to certain psychological characteristics and resources, including resilience and coping strategies, which have been associated with FCR [36]. Additionally, participation in these associations may enhance access to instrumental, emotional, and informational support, as well as disease engagement through interactions with professionals and the exchange of experiences with other survivors. Consequently, the relatively high levels of coping self-efficacy and perceived social support observed in the present study may partially reflect the support available within these associations. Participants were recruited from seven cancer survivor associations therefore the findings should be interpreted within the Croatian context, as cultural and healthcare-related factors may influence experiences of FCR. Additionally, the use of a convenience sample comprising 110 participants, the majority of whom were breast cancer survivors, further limits the generalizability of the findings to the broader population of breast and gynecological cancer survivors. These limitations highlight the need for future research using larger, probability-based samples. The wide range of time since diagnosis may have made it more difficult for some participants to accurately recall past information and experiences, potentially introducing recall bias. The sample predominantly consisted of older women diagnosed with early-stage cancer approximately a decade prior to participation in the study. However, the sample also included women who were not in remission and women with Stage IV disease. For these participants, cancer-related concerns captured by the FCRI-SF may have reflected fear of progression (FoP) rather than recurrence. Although FCR and FoP are closely related, evidence suggests that they are not equivalent constructs and should be considered separately [60]. Therefore, future studies should assess FCR and FoP separately using measures appropriate to participants’ clinical characteristics. Future research should also aim to include younger women, those with gynecological cancers and in the early post-treatment phase, as they may be more vulnerable to elevated and persistent FCR and report greater need for psychological support [14,53]. The presence of psychiatric disorders was assessed based on self-report rather than clinical evaluation, which may have limited the accuracy of this information. Additionally, excluding participants with reported psychiatric disorders may have reduced the external validity of the findings and resulted in lower estimates of psychological distress. This study did not consider physical comorbidities beyond the primary cancer diagnosis, which may further limit the generalizability of the findings. Future research should investigate the role of psychological and physical comorbidities, given their potential associations with FCR [61]. The present study focused only on a subset of the predisposing factors included in the revised Lee-Jones model of fear of cancer recurrence [34]. Future research could therefore extend this work by examining other components of the model, such as the role of healthcare professionals and the presence of cancer-related somatic symptoms. Furthermore, several authors have suggested that FCR could be associated with individuals’ beliefs about the curability or chronicity of cancer rather than by their actual medical status [33,62]. Consequently, subjective illness perceptions may represent an important avenue for future research and could provide further insight into the cognitive mechanisms underlying fear of cancer recurrence.

## 5. Conclusions

The process of psychological adjustment following a cancer diagnosis often continues well beyond primary treatment completion, with fear of cancer recurrence representing one of the most prominent unmet supportive care needs in survivorship. The findings of the present study indicate that more than half of the cancer survivors included in this study screen positive for clinically significant levels of FCR, across the wide range of time since diagnosis represented in the sample. Findings suggest that cognitive processing of cancer-related experiences and survivors’ confidence in their ability to cope with illness-related challenges may be relevant to FCR. They also point to cognitive processing and coping self-efficacy as potentially relevant areas for future studies. Further longitudinal research involving broader samples of breast and gynecological cancer survivors is warranted, with consideration to younger women in the early post-treatment period.

## Figures and Tables

**Table 1:** Participants’ demographic and clinical characteristics (*N* = 110).

Characteristic	Category	f (%)/*M*(*SD*)	Min–Max
Age	Years	61.3 (11.6)	31–87
Education level	Elementary school	5 (4.55)	
	High school	60 (54.6)	
	Bachelor’s degree	21 (19.1)	
	Master’s degrees	24 (21.8)	
Employment status	Employed and working	27 (24.55)	
	Employed, on sick leave	15 (13.6)	
	Unemployed	5 (4.55)	
	Retired	63 (57.3)	
Relationship status	Single	6 (5.45)	
	Married	63 (57.3)	
	Co-habiting/unmarried	6 (5.45)	
	Divorced	16 (14.5)	
	Widow	18 (16.4)	
	I’d rather not say	1 (0.9)	
Cancer type	Breast cancer	96 (87.3)	
	Gynecological cancer	13 (11.8)	
	Both	1 (0.9)	
Time since diagnosis	Months	104.3 (99.7)	2–495
Stage	In situ	15 (13.6)	
	I	53 (48.2)	
	II	13 (11.82)	
	III	14 (12.73)	
	IV	3 (2.73)	
	Don’t know	12 (10.9)	
Characteristic	Category	f (%)/*M*(*SD*)	min–max
^a^ Primary treatment	Surgical treatment	95 (86.4)	
	Chemotherapy	66 (60.0)	
	Radiotherapy	62 (56.4)	
	Hormonal therapy	40 (36.4)	
	Targeted therapy	21 (19.1)	
	Immunotherapy	6 (5.5)	
Metastases	No	78 (70.91)	
	Yes, lymph nodes	27 (24.55)	
	Yes, bones	2 (1.82)	
	Yes, digestive system	2 (1.82)	
	Yes, lungs	1 (0.9)	
Family history	No	57 (51.82)	
	Yes, mother	21 (19.1)	
	Yes, sister	9 (8.18)	
	Yes, grandma	8 (7.3)	
	Yes, aunt	13 (11.8)	
	Other	2 (1.8)	
Current remission status	No	21 (19.1)	
	Yes	72 (65.5)	
	Don’t know	17 (15.5)	
Currently receiving cancer-related treatment	No	51 (46.4)	
	Yes	54 (49.1)	
	Missing	5 (4.5)	
Time since the most recent oncologist visit	Months	8.7 (16.3)	0.03–118.3

f, frequency; *M* = mean; *SD* = standard deviation; ^a^, multiple responses were possible for treatment modalities; therefore, percentages do not sum to 100%.

**Table 2:** Descriptive statistics for observed variables (*N* = 110).

	*M* (*SD*)	C (Min, Max)	S-W	Skew	Kurt
FCR-SF	15.3 (7.7)	15 (0–33)	0.98	0.22	−0.76
IES-total	27.7 (16.8)	29 (0–73)	0.98	0.25	−0.37
Intrusion	10.9 (8.7)	8 (0–33)	0.93 **	0.67	−0.41
Avoidance	16.9 (10)	18 (0–40)	0.98 *	0.06	−0.70
Self-efficacy for coping	86 (15.5)	88.5 (42–108)	0.95 **	−0.68	−0.03
Social support- total	5.9 (1.3)	6. 2 (1.2–7)	0.81 **	−1.73	2.93
Support- family	5.9 (1.5)	6.5 (1–7)	0.73 **	−1.77	2.50
Support- friends	5.6 (1.5)	6 (1.3–7)	0.85 **	−1.17	0.72
Support- significant other	6 (1.5)	6.8 (1–7)	0.71 **	−2.04	4.00

* *p* < 0.05; ** *p* < 0.01; FCR-SF, Fear of cancer recurrence; IES, Impact of Event Scale total score; *M*, mean; *SD*, standard deviation; C, median; S-W, Shapiro–Wilk statistics; Skew, skewness; Kurt, kurtosis.

**Table 3:** Correlation analysis of the observed variables (*N* = 110).

	1	2	3	4	5	6	7	8	9	10	11	12
(1) FCR- SF	-											
(2) Age	−0.26 **											
(3) Time since diagnosis	−0.16	0.44 **										
(4) Chemotherapy	0.22 *	−0.16	0.03									
(5) Time since last examination	−0.13	0.24 *	0.35 **	0.07								
(6) Intrusion	0.56 **	−0.15	−0.24 *	0.01	−0.12							
(7) Avoidance	0.39 **	−0.16	−0.20 *	0.15	−0.03	0.63 **						
(8) Self-efficacy for coping	−0.41 **	0.06	0.02	−0.07	−0.11	−0.41 **	−0.38 **					
(9) Social support- total	−0.19	−0.09	0.04	0.06	0.10	−0.15	−0.28 **	0.35 **				
(10) Support- family	−0.19	0.02	0.01	−0.06	0.02	−0.15	−0.23 *	0.33 **	0.81 **			
(11) Support- friends	−0.11	−0.14	0.11	0.15	0.18	−0.07	−0.17	0.15	0.80 **	0.35 **		
(12) Support- significant other	−0.17	−0.13	−0.00	0.07	0.05	−0.17	−0.29 **	0.40 **	0.91 **	0.64 **	0.67 **	-

* *p* < 0.05; ** *p* < 0.01; FCR-SF, Fear of cancer recurrence.

**Table 4:** Hierarchical regression analysis predicting fear of cancer reoccurrence (*N* = 102).

	Model 1						Model 2						Model 3					
	*B*	*SE*	ß	*t*	*p*	95% CI	*B*	*SE*	ß	*t*	*p*	95% CI	*B*	*SE*	ß	*t*	*p*	95% CI
Variables																		
Age	−0.14	0.07	−0.21	−2.10 *	0.04	[−0.27, −0.01]	−0.09	0.06	−0.13	−1.57	0.12	[−0.20, 0.02]	−0.09	0.06	−0.14	−1.71	0.09	[−0.20, −0.02]
Time_diagnosis	−0.002	0.002	−0.10	−1.01	0.31	[−0.01, 0.002]	−0.001	0.001	−0.05	−0.67	0.51	[−0.003, 0.001]	−0.001	0.001	−0.06	−0.94	0.35	[−0.003, −0.001]
Chemotherapy	3.56	1.54	0.22	2.32 *	0.02	[0.51, 6.61]	3.78	1.28	0.24	2.96 **	0.004	[1.24, 6.33]	3.88	1.25	0.24	3.11 **	0.002	[1.41, 6.35]
Intrusion							0.50	0.09	0.56	5.57 **	<0.001	[0.32, 0.68]	0.45	0.09	0.50	4.87 **	<0.001	[0.26, 0.63]
Avoidance							−0.01	0.08	−0.02	−0.14	0.89	[−0.17, 0.15]	−0.06	0.08	−0.07	−0.73	0.47	[−0.22, 0.10]
Self-efficacy for coping													−0.10	0.04	−0.20	−2.27 *	0.03	[−0.19, −0.01]
Social support													−0.54	0.56	−0.08	−0.96	0.34	[−1.66, 0.58]
*R* ^2^	0.123						0.418						0.466					
Adjusted *R*^2^	0.096						0.388						0.426					
Δ*R*^2^	0.123						0.296						0.047					
*F*	4.616 **						13.957 **						11.830 **					

* *p* < 0.05; ** *p* < 0.01; *B*, unstandardized regression coefficient; ß, standardized regression coefficient; *SE*, standard error; *t*, t-statistic; *R*^2^, coefficient of determination; Adjusted *R*^2^, adjusted coefficient of determination; Δ*R*^2^, change in coefficient of determination; F, F statistic; FCR, Fear of cancer recurrence; time_diagnosis, time since diagnosis; social support, social support_total.

## Data Availability

The authors have saved all the data supporting the conclusions in this research. Any valid request will be granted access to data from the corresponding author.

## Supplementary Materials

The following supporting information can be downloaded at: https://www.mdpi.com/article/10.3390/healthcare14172722/s1, Table S1: STROBE Statement—Checklist of items that should be included in reports of cross-sectional studies.
